# Supplementary material for: De novo acquisition of antibiotic resistance in six species of bacteria
Source: Microbiol Spectr. 2025 Feb 5;13(3):e01785-24. doi: 10.1128/spectrum.01785-24 (PMC11878088; doi:10.1128/spectrum.01785-24)
Supplement: Supplemental material — Fig. S1 to S4; Tables S1 to S4. [file spectrum.01785-24-s0001.docx]

***De novo* acquisition of antibiotic resistance in six *species of bacteria***

**Supplementary Figures and Tables**

**Supplementary Method**  Data processing of Whole genome sequencing………………………………………....2

**Supplementary Figures S1** The schema of adaptive antibiotic evolution……….3

**Supplementary Figures S2** Trajectory of Minimal inhibitory concentration for six species………………………………………….4

**Supplementary Figures S3**  The distribution of allele frequency at the first measurement………………………………………10

**Supplementary Figures S4** The allele frequency change between the two time measurements……………………………………...11

**Supplementary Table S1** The Amino acid change observed in β-lactam evolution……………………………………………1

**Supplementary Table S2** The Amino acid change observed in enrofloxacin evolution……………………………...2

**Supplementary Table S3** The Amino acid change observed in kanamycin evolution……………………………......3

**Supplementary Table S4** Mutator genotype involved in antibiotic evolution…4

**Data processing of Whole genome sequencing**

Data processing was performed using Snakemake(Mölder et al., 2021).

The quality of the raw sequences was assessed using fastQC6 (2). Reads were trimmed with Trimmomatic (3) for removal of Illumina adapters and sequences that did not meet the set criteria of 2:30:10:1 SLIDINGWINDOW:4:20 MINLEN:50. After trimming, the reads were once again subjected to quality control with fastQC. Reads were deduplicated using Clumpify (4) and mapped to the reference genome using BWA mem (Li 2013). The following reference genomes have been used: RefSeq GCA_000009045.1 (*B. subtilis* 168), GCA_000172575.2 (*E. faecalis*), GCA_021383745.1 (*S. enterica subsp. houtenae*), GCA_019551355.1 (*S. aureus*) and GCA_901472495.1 (*Y. enterocolitica*). There was no available reference genome ~~was~~ for the *A. pittii* strain used in this study. The reference genome for this strain was de novo assembled with SPAdes genome assembler (6) with settings --isolate -k 21,33,55,77,99,121 --cov-cut-off auto. The de novo assembly was scaffolded with RagTag (7)using minimap2 as the aligner and annotated with Prokka (8). After mapping, read group sample names were added to the bam files with picard AddOrReplaceReadGroups and duplicates were marked with gatk MarkDuplicatesSpark. Variant calling was performed with both freebayes(8) and LoFreq (Wilm et al. 2012). Freebayes was executed without incorporating prior information of variant calls or copy number variation. The following settings were used: -F 0.01 -C 1 -X -p 1 -u -g 1000 --pooled-continuous. The allele frequency (AF) in the VCF output was replaced with AO/(AO+DP) using a custom script to obtain a more sensible value for samples processed with the --pooled-continuous option. For LoFreq, the bam files were preprocessed by indel realignment and base quality score recalibration. Indel realignment was performed with the RealignerTargetCreator and IndelRealigner tools of GenomeAnalysisTK (version 3.5-0-g36282e4) and base quality score recalibration was performed with the BaseRecalibrator and ApplyBQSR tools of GATK(9). For the BaseRecalibrator tool, the VCF output of Freebayes filtered with “QUAL > 70” was used as a high-confidence variant call file using the --known-sites option and the --bqsr-baq-open-penalty was set to 30. The VCF output of Freebayes and LoFreq was filtered with a quality score threshold of 100 and normalized by decomposing multi-allelic variants, left-alignment of variants and the removal of superfluous nucleotides to reach parsimony. Any variants containing “N”, indicative of inclusion of scaffolding sequence, were filtered out as well. The VCF output was merged using a custom script. Variant annotation was performed with SnpEff. An SnpEff database was built with the buildDbNcbi.sh script for all species except *Y. enterocolitica*, for which there was a pre-existent database. For the *S. enterica* and *S. aureus* samples, a plasmid database was created in addition. After annotation, descriptions of gene function were added from the respective GBK files to the VCF headers using a custom script. The annotated VCF files were filtered for variants that were present in the control samples. The allele frequencies of the VCF files were extracted and plotted using a custom script. A cutoff value for allele frequency was determined using these plots and applied with the aim of excluding noise and biologically irrelevant variants. The VCF files were then categorized into three categories per measurement timepoint using bcftools isec: all variants found in the timepoint (1), variants found exclusively in the timepoint (2) and variants that were found in both timepoints (3). Exact variants were considered in forming the three categories. The allele frequency changes for variants in the third category were computed and plotted using a custom script.

**Figure S1 A) Six species are exposed in six antibiotic evolutions. B) The schema of adaptive antibiotic evolution protocol**
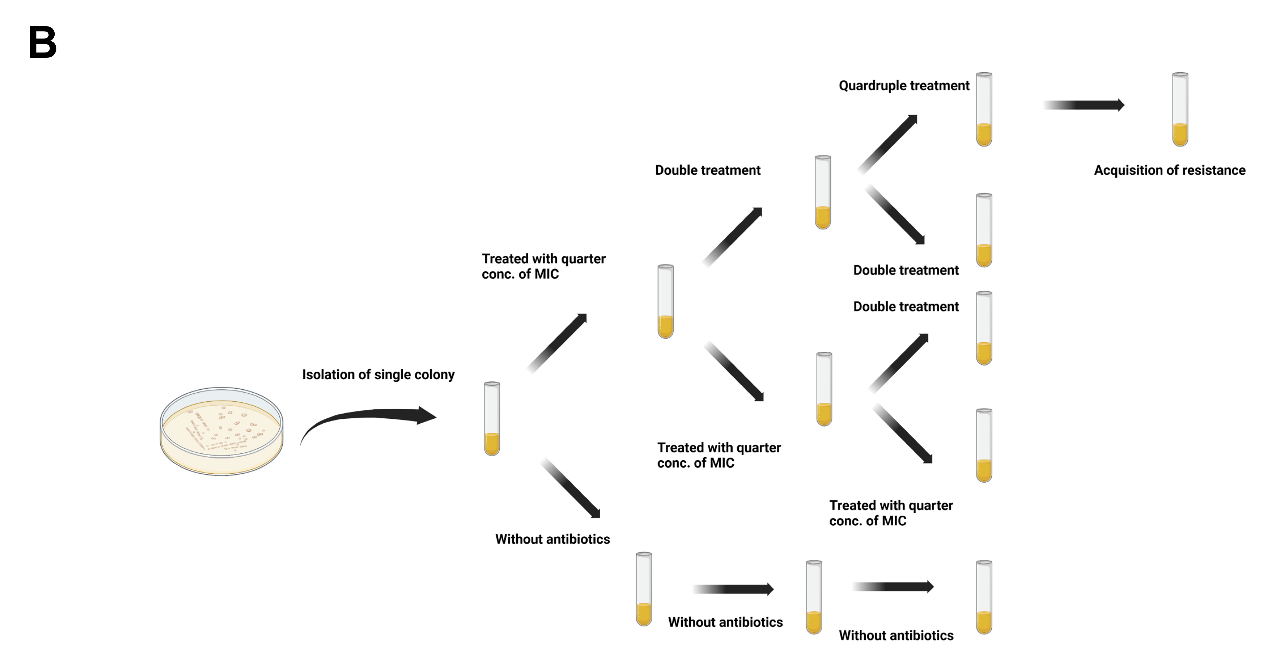


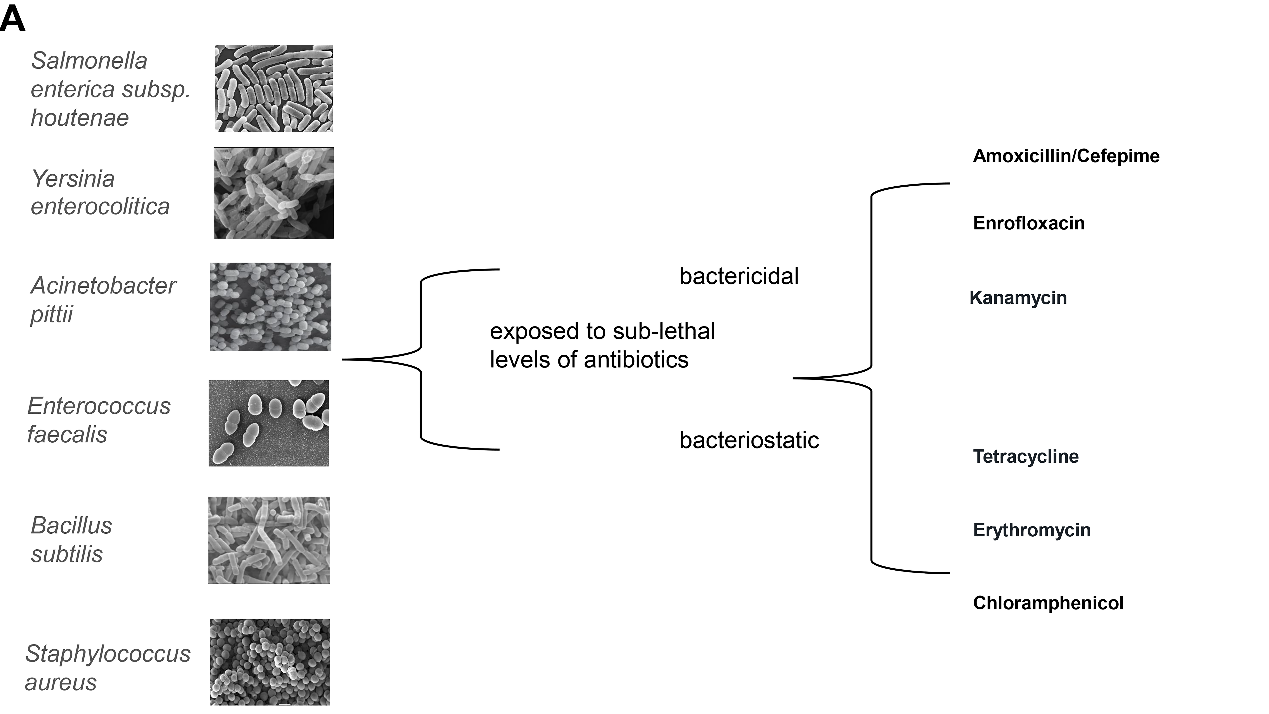


**
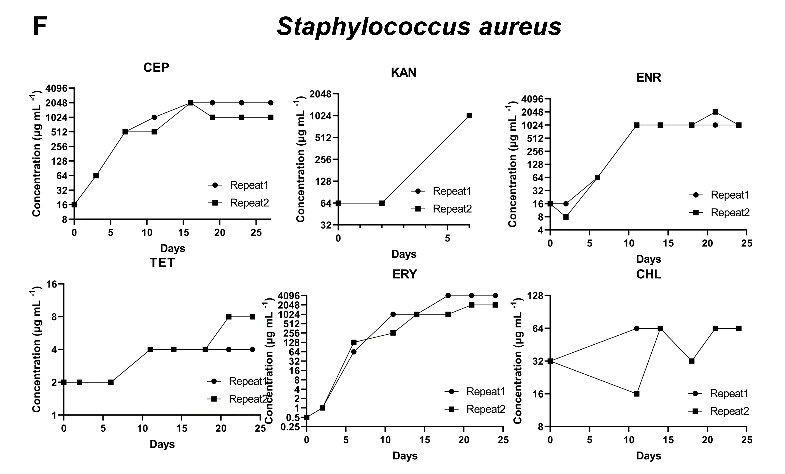

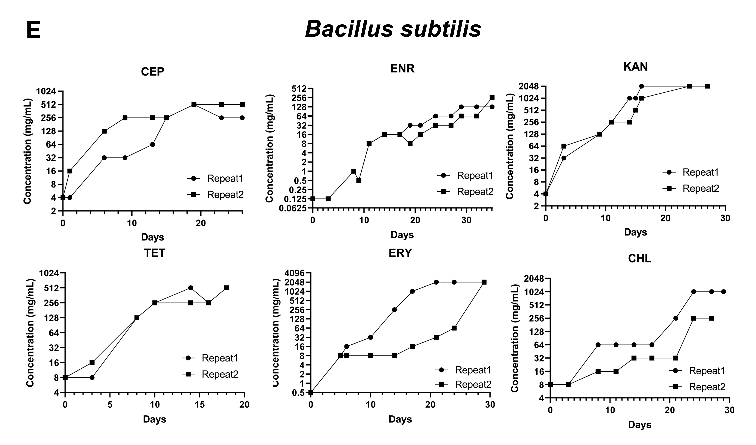

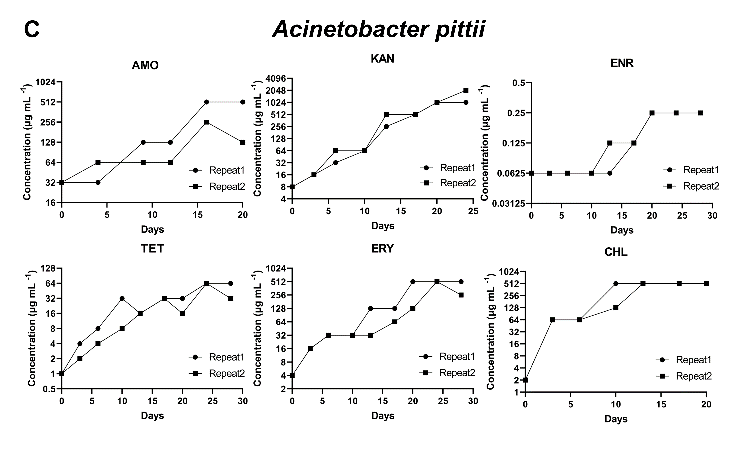

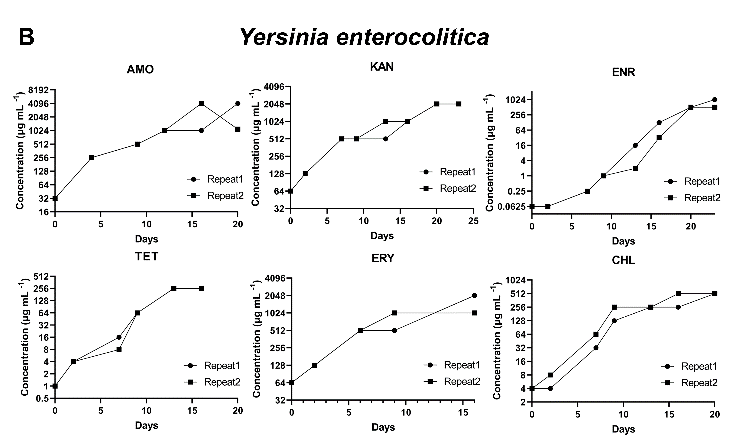

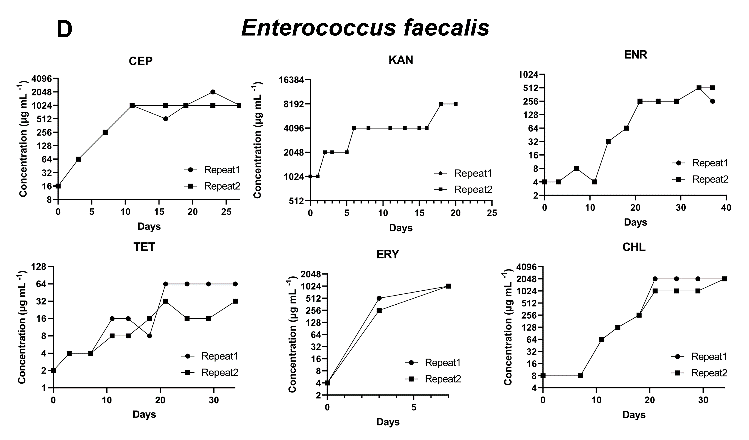
**
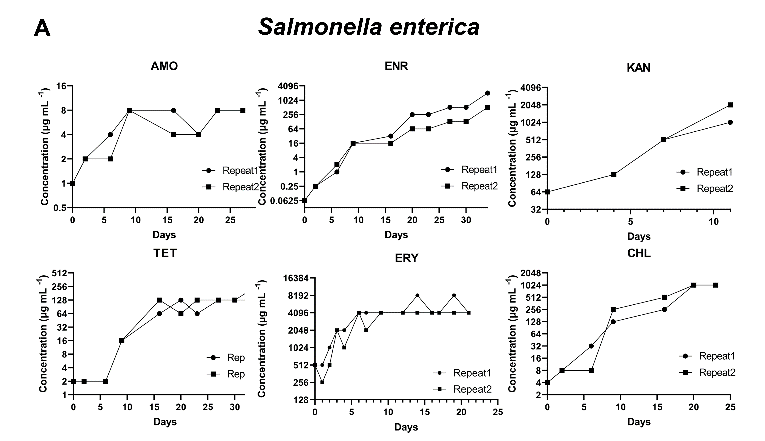
**Figure S2 Trajectory of Minimal inhibitory concentration for six species treated with six antibiotics.**

**
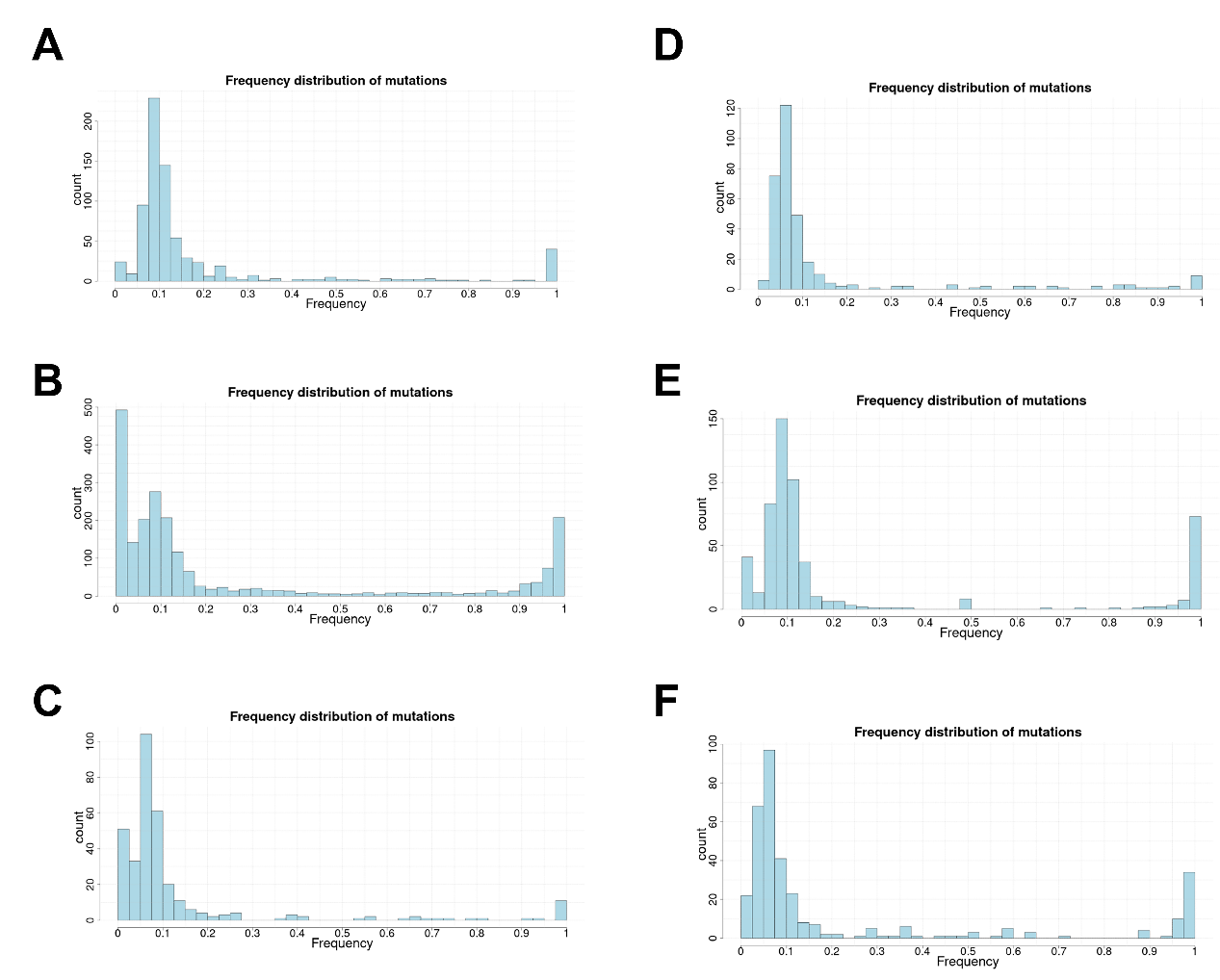
Figure S3**

**Figure S3 The distribution of allele frequency at the first measurement** A) *S. enterica*, B) *Y. enterocolitica*, C) *A. pittii*, D) *E. faecalis*, E) *B. subtilis*, F) *S. aureus*;

**
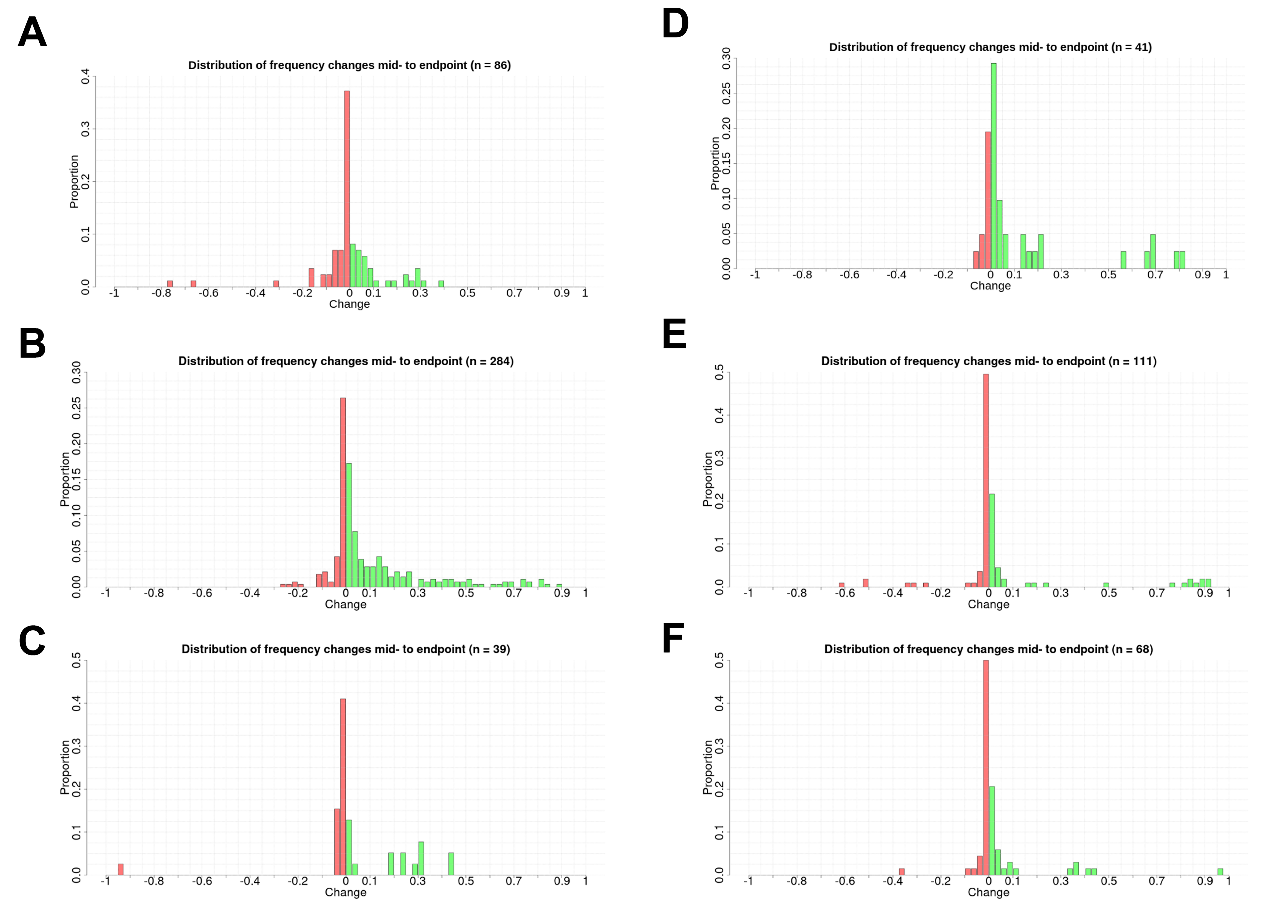
Figure S4**

**Figure S4** **The allele frequency change between the two timepoint** A) *S. enterica*, B) *Y. enterocolitica*, C) *A. pittii*, D) *E. faecalis*, E) *B. subtilis*, F) *S. aureus*; Allele frequency was defined as the count of full observations of the alternate haplotype divided by the total read depth at the locus. Bars in the negative-value range are colored red, and bars in the positive-value range are colored green.

**Table S1 Amino acid changes observed in amoxicillin or cefepime evolution**

| **Species** | **Gene** | **mutation type** | **Timepoint** | **Amino acid change** | **Allele frequency** |
| --- | --- | --- | --- | --- | --- |
| ***S. enterica*-1** | ***pbp2*** | **Missense_variant** | **1 \| 2** | **Glu592Gly** | **1 \| 1** |
| ***S. enterica*-2** | ***pbp 2*** | **Missense_variant** | **1 \| 2** | **Glu592Gly** | **1 \| 0.998** |
| ***A. pittii* -1** | ***ampC*** | **Promoter Mutation** | **2** | **c.-89G>A** | **1** |
| ***A. pittii*-2** | ***ampC*** | **Promoter Mutation** | **1 \| 2** | **c.-89G>A** | **1** |
| ***B. subtilis*-1** | ***pbpH*** | **Frame shift** | **1 \| 2** | **Ser296fs** | **0.958** |
|  | ***pbpC*** | **Missense_variant** | **1** | **Pro414Leu** | **0.869** |
|  | ***pbpC*** | **Missense_variant** | **2** | **Leu462Ser** | **0.320** |
|  | ***pbpC*** | **Missense_variant** | **2** | **Ser594Leu** | **1** |
| ***B. subtilis*-2** | ***pbpH*** | **Frame shift** | **1 \| 2** | **Thr651fs** | **0.889 \| 0.958** |
|  | ***pbpC*** | **Missense_variant** | **1** | **Pro414Leu** | **0.973** |
|  | ***pbpD*** | **Frame shift** | **1** | **Glu303fs** | **0.915** |
|  | ***pbpX*** | **Frame shift** | **2** | **Ser318fs** | **0.943** |
|  | ***pbpC*** | **Missense_variant** | **2** | **Ala612Pro** | **1** |
| ***S. aures*-1** | ***pbp*** | **Missense_variant** | **2** | **Leu599Ser** | **0.843** |

**Table S2 Amino acid changes observed in enrofloxacin evolution**

| **Species** | **Gene** | **mutation type** | **Timepoint** | **Amino acid change** | **Allele frequency** |
| --- | --- | --- | --- | --- | --- |
| ***S. enterica*-1** | ***gyrA*** | **Missense_variant** | **1 \| 2** | **Ser83Phe** | **1 \| 0.980** |
|  | ***gyrA*** | **Missense_variant** | **1 \| 2** | **Asp87Gly** | **1 \| 0.975** |
|  | ***parC*** | **Missense_variant** | **1** | **Ser80Phe** | **1** |
|  | ***parC*** | **Missense_variant** | **2** | **Asp79Asn** | **0.988** |
|  | ***parC*** | **Missense_variant** | **2** | **Ser80Arg** | **0.985** |
| ***S. enterica*-2** | ***gyrA*** | **Missense_variant** | **1 \| 2** | **Asp87Asn** | **1 \| 1** |
|  | ***gyrA*** | **Missense_variant** | **2** | **Ser83Phe** | **1** |
|  | ***parC*** | **Missense_variant** | **1** | **Ser80Phe** | **1** |
|  | ***parC*** | **Missense_variant** | **2** | **Ser80Arg** | **0.994** |
| ***Y. enterocolitica*-1** | ***gyrA*** | **Missense_variant** | **1 \| 2** | **Ser83Ile** | **1 \| 1** |
|  | ***gyrA*** | **Missense_variant** | **2** | **Asp87Asn** | **0.991** |
|  | ***gyrA*** | **Missense_variant** | **2** | **Val54Ala** | **0.129** |
|  | ***parC*** | **Missense_variant** | **2** | **Gly82Asp** | **1** |
| ***Y. enterocolitica*-2** | ***gyrA*** | **Missense_variant** | **1 \| 2** | **Ser83lle** | **0.978 \| 1** |
|  | ***parC*** | **Missense_variant** | **1** | **Pro573Leu** | **0.117** |
|  | ***parC*** | **Missense_variant** | **1** | **Glu88Lys** | **0.072** |
|  | ***parC*** | **Missense_variant** | **2** | **Gly82Asp** | **0.992** |
| ***A. pittii*-1** | ***gyrA*** | **Missense_variant** | **1 \| 2** | **Asp70Gly** | **0.674 \| 0.991** |
|  | ***gyrA*** | **Missense_variant** | **2** | **Ser81Leu** | **0.922** |
| ***A. pittii*-2** | ***parC*** | **Missense_variant** | **1** | **Asn297Thr** | **0.0704** |
| ***E. faecalis-*1** | ***gyrA*** | **Missense_variant** | **1** | **Ser84Arg** | **0.523** |
|  | ***gyrA2*** | **Missense_variant** | **1** | **Ser85Arg** | **0.577** |
|  | ***gyrA2*** | **Missense_variant** | **1 \| 2** | **Ser85Ile** | **0.426 \| 1** |
|  | ***gyrA*** | **Missense_variant** | **2** | **Ser84Ile** | **0.999** |
| ***E. faecalis*-2** | ***gyrA*** | **Missense_variant** | **1 \| 2** | **Ser84Arg** | **0.994 \| 0.998** |
|  | ***gyrA2*** | **Missense_variant** | **1 \| 2** | **Ser85Arg** | **1 \| 1** |
|  | ***gyrA2*** | **Missense_variant** | **2** | **Glu89Gly** | **0.679** |
|  | ***gyrA2*** | **Missense_variant** | **2** | **Glu89Lys** | **0.272** |
| ***B. subtilis*-1** | ***gyrA*** | **Missense_variant** | **1 \| 2** | **Ser84Leu** | **1 \| 1** |
|  | ***gyrA*** | **Missense_variant** | **1 \| 2** | **Glu88Gly** | **1 \| 1** |
|  | ***parC*** | **Missense_variant** | **1 \| 2** | **Ser82Ile** | **1 \| 1** |
|  | ***parC*** | **Missense_variant** | **2** | **Glu86Lys** | **1** |
| ***B. subtilis*-2** | ***gyrA*** | **Missense_variant** | **1 \| 2** | **Ser84Leu** | **1 \| 0.995** |
|  | ***parC*** | **Missense_variant** | **1 \| 2** | **Ser82Ile** | **1 \| 1** |
| ***S. aureus*-1** | ***gyrA*** | **Missense_variant** | **1 \| 2** | **Ser84Leu** | **1 \| 0.999** |
|  | ***parC*** | **Missense_variant** | **2** | **Ser80Phe** | **0.999** |
|  | ***parC*** | **Missense_variant** | **2** | **Glu84Lys** | **0.999** |
| ***S. aureus*-2** | ***gyrA*** | **Missense_variant** | **1 \| 2** | **Ser84Leu** | **1 \| 0.997** |
|  | ***parC*** | **Missense_variant** | **2** | **Ser80Phe** | **0.999** |
|  | ***parC*** | **Missense_variant** | **2** | **Glu84Lys** | **0.831** |
|  | ***parC*** | **Missense_variant** | **2** | **Glu84Gln** | **0.169** |

**Table S3 Amino acid changes observed in kanamycin evolution**

| **Species** | **Gene** | **mutation type** | **Timepoint** | **Amino acid change** | **Allele frequency** |
| --- | --- | --- | --- | --- | --- |
| ***S. enterica-1*** | ***fusA*** | **Missense_variant** | **1** \| **2** | **Ala608Val** | **1 \| 1** |
|  | ***fusA*** | **Missense_variant** | **2** | **Phe601Val** | **1** |
| ***S. enterica*-2** | ***fusA*** | **Missense_vaiiant** | **1 \| 2** | **Thr668Ala** | **0.25 \| 0.555** |
|  | ***fusA*** | **Missense_vaiiant** | **1** | **Phe605Val** | **0.64** |
|  | ***fusA*** | **Missense_variant** | **2** | **Ala493Gly** | **0.361** |
|  | ***fusA*** | **Missense_variant** | **2** | **Ser589Ala** | **0.561** |
| ***Y. enterocolitica*-1** | ***fusA*** | **Missense_vaiiant** | **1 \| 2** | **Ala590Val** | **0.628 \| 1** |
|  | ***fusA*** | **Missense_vaiiant** | **1 \| 2** | **Phe603leu** | **0.307 \| 1** |
| ***Y. enterocolitica*-2** | ***fusA*** | **Missense_vaiiant** | **1** | **Phe603Leu** | **0.565** |
|  | ***fusA*** | **Missense_vaiiant** | **2** | **Ala590Val** | **0.923** |
| ***A. pittii*-1** | ***fusA*** | **Missense_variant** | **1** | **Gly148Cys** | **0.949** |
|  | ***fusA*** | **Missense_variant** | **2** | **Phe535lle** | **1** |
| ***A. pittii*-2** | ***fusA*** | **Missense_variant** | **1** | **Ala595Val** | **1** |
|  | ***fusA*** | **Missense_variant** | **2** | **Phe535lle** | **1** |
| ***E. faecalis-*1** | ***fusA*** | **Missense_variant** | **1 \| 2** | **Gly665Cys** | **0.948 \| 1** |
| ***E. faecalis-*2** | ***fusA*** | **Missense_variant** | **1** | **Ala539Glu** | **0.427** |
|  | ***fusA*** | **Missense_variant** | **1** | **Ala660Glu** | **0.141** |
|  | ***fusA*** | **Missense_variant** | **1** | **Asp572Gly** | **0.062** |
|  | ***fusA*** | **Missense_variant** | **1 \| 2** | **Gly665Cys** | **0.2085 \| 1** |
| ***B. subtilis*-1** | ***fusA*** | **Missense_variant** | **1 \| 2** | **Val86Ala** | **0.993 \| 1** |
| ***B. subtilis*-2** | ***fusA*** | **Missense_variant** | **2** | **Gly551Cys** | **1** |
| ***S. aureus*-1** | ***fusA*** | **Missense_variant** | **1** | **Gly507Ser** | **1** |
|  | ***fusA*** | **Frame shift** | **2** | **Ser481dup** | **0.980** |
| ***S. aureus*-2** | ***fusA*** | **Missense_variant** | **2** | **Ala580Val** | **0.85** |

**Table S4 Mutator genotype involved in antibiotic evolution**

|  | **Allel frequency** | **Gene** | **Mutation type** | **Amino acid change** | **Base**  **change** | **Mutation**  **number** |
| --- | --- | --- | --- | --- | --- | --- |
| ***S. enterica*-ENR-F-1** | **0.97** | ***mutS*** | **Frameshift** | **Ala440fs** | **C. 1318delG** | **187** |
| ***A. pittii*-ENR-M-1** | **0.64** | ***mutL*** | **Frameshift** | **Glu215fs** | **C. 636_642dupTAGCGGA** | **47** |
| ***A. pittii*-ENR-F-1** | **1** | ***mutL*** | **Frameshift** | **Glu215fs** | **C. 636_642dupTAGCGGA** | **61** |
| ***B. subtilis*-CEF-M-2** | **1** | ***mutL*** | **Frameshift** | **Val362fs** | **C. 1083delA** | **90** |
| ***B. subtilis*-CEF-F-2** | **0.96** | ***mutL*** | **Frameshift** | **Val362fs** | **C. 1083delA** | **240** |
| ***B. subtilis*-CEF-M-1** | **1** | ***mutS*** | **Missense** | **Val65Ala** | **C. 194 T>C** | **68** |
| ***B. subtilis*-CEF-F-1** | **1** | ***mutS*** | **Missense** | **Val65Ala** | **C. 194 T>C** | **136** |
| ***B. subtilis*-CEF-F-1** | **1** | ***mutS*** | **Missense** | **Arg189Leu** | **C. 556 G>T** | **136** |

M: Evolved populations were sequenced in half evolution: F: Evolved populations were sequenced in final evolution.

**Reference**

1. Mölder F, Jablonski KP, Letcher B, Hall MB, Tomkins-Tinch CH, Sochat V, Forster J, Lee S, Twardziok SO, Kanitz A, Wilm A, Holtgrewe M, Rahmann S, Nahnsen S, Köster J. 2021. Sustainable data analysis with Snakemake. F1000Res 10.

2. Andrews S. 2010. FastQC - A quality control tool for high throughput sequence data. http://www.bioinformatics.babraham.ac.uk/projects/fastqc/. Babraham Bioinformatics.

3. Bolger AM, Lohse M, Usadel B. 2014. Trimmomatic: A flexible trimmer for Illumina sequence data. Bioinformatics 30.

4. Bushnell B. 2012. BBMap: A Fast, Accurate, Splice-Aware Aligner. BMC Bioinformatics 13.

5. Li H. 2013. [Heng Li - Compares BWA to other long read aligners like CUSHAW2] Aligning sequence reads, clone sequences and assembly contigs with BWA-MEM. arXiv preprint arXiv.

6. Prjibelski A, Antipov D, Meleshko D, Lapidus A, Korobeynikov A. 2020. Using SPAdes De Novo Assembler. Curr Protoc Bioinformatics 70.

7. Alonge M, Lebeigle L, Kirsche M, Jenike K, Ou S, Aganezov S, Wang X, Lippman ZB, Schatz MC, Soyk S. 2022. Automated assembly scaffolding using RagTag elevates a new tomato system for high-throughput genome editing. Genome Biol 23.

8. Garrison E, Marth G. 2012. Haplotype-based variant detection from short-read sequencing -- Free bayes -- Variant Calling -- Longranger. arXiv preprint arXiv:12073907.

9. Van der Auwera GA, Carneiro MO, Hartl C, Poplin R, del Angel G, Levy-Moonshine A, Jordan T, Shakir K, Roazen D, Thibault J, Banks E, Garimella K V., Altshuler D, Gabriel S, DePristo MA. 2013. From fastQ data to high-confidence variant calls: The genome analysis toolkit best practices pipeline. Curr Protoc Bioinformatics https://doi.org/10.1002/0471250953.bi1110s43.
